# De novo three-way chromosome translocation 46,XY,t(4;6;21)(p16;p21.1;q21) in a male with cleidocranial dysplasia

**DOI:** 10.1002/ajmg.a.31750

**Published:** 2008-02-15

**Authors:** Smita M Purandare, Roberto Mendoza-Londono, Svetlana A Yatsenko, Dobrawa Napierala, Daryl A Scott, Tarek Sibai, Kari Casas, Patrick Wilson, Jiyun Lee, Razia Muneer, Joe C Leonard, Faridali G Ramji, Ralph Lachman, Shibo Li, Pawel Stankiewicz, Brendan Lee, John J Mulvihill

**Affiliations:** 1 Section of Genetics, Department of Pediatrics, University of Oklahoma Health Sciences CenterOklahoma City, Oklahoma; 2 Department of Molecular and Human Genetics, Baylor College of MedicineHouston, Texas; 3 Division of Clinical and Metabolic Genetics, The Hospital for Sick ChildrenToronto, Canada; 4 Department of Radiological Sciences, University of Oklahoma Health Sciences CenterOklahoma City, Oklahoma; 5 International Skeletal Dysplasia Registry, Cedars Sinai Medical CenterLos Angeles California; 6 Howard Hughes Medical InstituteChevy Chase, Maryland

**Keywords:** three-way chromosome translocation, cleidocranial dysplasia (CCD), fluorescence in situ hybridization (FISH), phenotype–genotype correlation

## Abstract

Cleidocranial dysplasia (CCD) is an autosomal dominant skeletal dysplasia associated with cranial, clavicular, and dental anomalies. It is caused by mutations in the *RUNX2* gene, which encodes an osteoblast-specific transcription factor and maps to chromosome 6p21. We report clinical and molecular cytogenetic studies in a patient with clinical features of CCD including wormian bones, delayed fontanel closure, hypoplastic clavicles and pubic rami, and supernumerary dentition. Additional abnormalities of bone growth and connective tissue, including easy bruisability, scarring, bleeding, joint hypermobility, and developmental delay were also observed. Molecular cytogenetic studies identified a de novo apparently balanced three-way translocation 46,XY,t(4;6;21)(p16;p21.1;q21). Further mapping revealed the breakpoint on 6p21 to be ∼50 kb upstream of exon 1 of the *RUNX2* gene, with *RUNX2* being intact on the derivative chromosome 6. We hypothesize that the proband's CCD has arisen from disruption of the developmentally regulated gene *RUNX2* at the 6p21 breakpoint, due to a position effect mutation which may have altered the expression of the gene. Further studies might unravel a new regulatory element for *RUNX2.*

## INTRODUCTION

Cleidocranial dysplasia (CCD: OMIM 119600) is an autosomal dominant skeletal dysplasia affecting bones derived from endochondral and intramembranous ossification. The diagnosis of CCD is based on clinical and radiological findings [Mendoza-Londono and Lee, 2006]. Hallmark features of CCD include large open fontanels with delayed or absent closure, midface hypoplasia, abnormal dentition including supernumerary teeth, clavicular hypoplasia, and hand abnormalities such as brachydactyly [Taybi and Lachman, 1996]. Intellectual development is usually normal in patients with CCD [Cooper et al., 2001]

Skeletal elements of patients with CCD show disordered regulation of endochondral and intramembranous ossification [Zheng et al., 2005]. Sixty to seventy percent of cases of CCD are caused by mutations in the gene encoding transcription factor *RUNX2* (runt-related transcription factor 2) located on chromosome 6p21 [Lee et al., 1997; Mundlos et al., 1997]. The mutations include missense, deletion, splice, insertion, and nonsense mutations [Zhou et al., 1999, Yoshida et al., 2002]. Microdeletions of the gene have also been reported [Gelb et al., 1995, Mundlos et al., 1995, Izumi et al., 2006]. *RUNX2* is a transcription factor essential for osteoblast differentiation and chondrocyte maturation during endochondral ossification [Zheng et al., 2005]. Heterozygote *RUNX2* mice display abnormalities similar to individuals with CCD [Shapiro, 1999]. The expression of *RUNX2* during development is regulated by at least two separate promoters. Additional enhancer elements upstream of the gene also modify its expression [Stock and Otto, 2005].

We present a patient with skeletal findings consistent with CCD and additional atypical findings including learning disability, developmental delay, and features suggestive of connective tissue disorders including easy bruising, joint laxity, and dislocations. We identified a complex translocation involving three chromosomes and carried out further molecular investigations to determine the integrity of the *RUNX2* gene on 6p21 (region involved in the translocation), which has been implicated in the causation of CCD.

## CLINICAL REPORT

A presently 17-year-old Caucasian male was recognized at 3 years of age to have a complex chromosome translocation involving chromosomes 4p, 6p, and 21q, while being evaluated for developmental delay and minor dysmorphisms including a large head, short stature, hypertelorism, and supernumerary dentition. No specific syndromic diagnosis was made. Nearly a decade later, he presented with severe joint pain, bleeding tendencies, and delayed puberty, and a clinical and cytogenetic re-evaluation was done.

In the interval he had polydontia requiring removal of supernumerary teeth, umbilical hernia repair, normal CT of the head at age 11 years, obstructive sleep apnea syndrome requiring tonsillectomy and adenoidectomy, and septoplasty (open reduction of nasal fracture with repair of septum). Due to recurrent epistaxis and left nasal polyps he had removal of the polyps, which showed histopathological features of developing angiofibromas. In addition to the large cranium, abnormal dentition, short stature, scoliosis, joint laxity, and recurrent respiratory infections, all features of CCD, he also had learning disability which is not a feature typical of CCD.

**Fig. 1:**
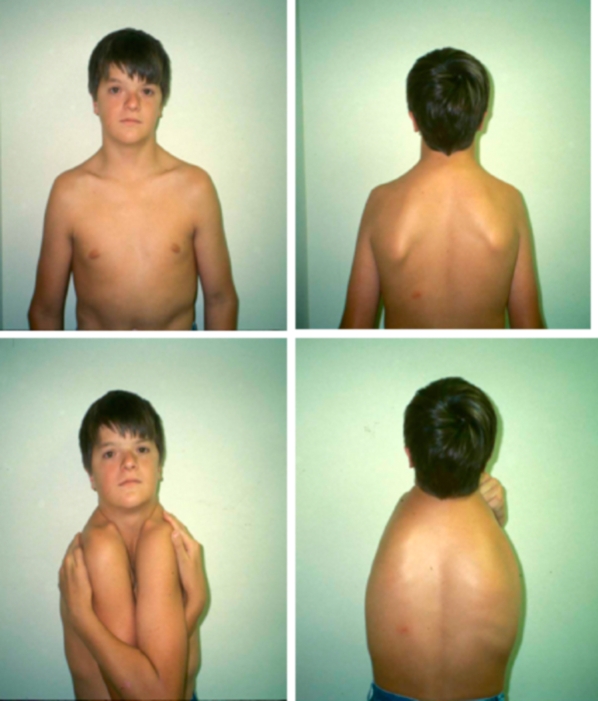
Clinical findings at age 14 years. [Color figure can be viewed in the online issue, which is available at www.interscience.wiley.com.]

Abnormalities of bone growth and connective tissue evolved over time. At 14 years of age (Fig. 1), he complained of easy bruisability, scarring, bleeding, joint pain, increased shoulder motion, and occasional shortness of breath during heavy exercise. Pubertal development was delayed. Height, weight, and head circumference were at the 25th, 50th, and 80th centiles, respectively. He had soft skin with multiple bruises. His face was asymmetric with leftward deviation of the nose. There was kyphosis with a slight rightward deviation. He had pes planus along with hypoplastic fourth and fifth toenails, bilaterally. He could actively dislocate his thumb, knees, and hip. He had bilateral contractures at the elbows, and two cafe-au-lait macules. Endocrine studies showed normal gonadotropin levels. At age 16 years, height was at the 20th centile, weight was at the 80th centile, and occipital–frontal circumference was at the 98th centile. Pubertal development was Tanner III for male pubic hair. Bilaterally there was decreased supination and extension of the elbows and shoulders. The clavicles are diminutive allowing excessive adduction of the shoulders.

**Fig. 2:**
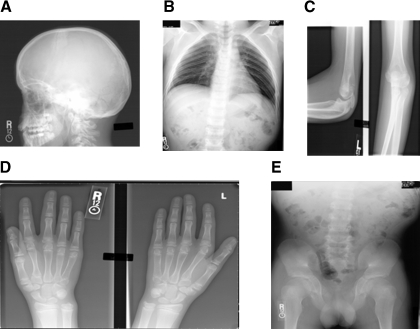
**A**: Wormian bones. **B**: Short clavicles. **C**: Dislocated radial head. **D**: Supernumerary-pseudoepiphyseal centers at the bases of the metacarpals 1, 2, and 5. **E**: Hypoplastic pubic bones and mild hypoplasia of the ischia.

Skeletal survey at age 14 years (Fig. 2) showed features consistent with CCD, including wormian bones, short clavicles, posterior wedging of vertebral bodies in the thoracic and lumbar spine, hypoplastic pubic bones and ischia, coxa valga, and long metacarpals and phalanges with supernumerary pseudoepiphyseal centers at the bases of the metacarpals one, two, and five.

## RESULTS

**Fig. 3:**
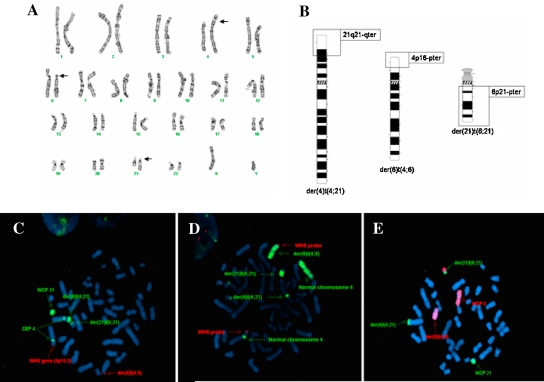
**A**: GTG-banding karyotype of lymphocytes of the patient. **B**: Idiogram of derivative chromosomes 4, 6, and 21, with their corresponding translocated segments. **C**: DNA probes corresponding to chromosome 4p (red) and whole chromosome painting probe for chromosome 21 (green) and hybridized to metaphase chromosomes and revealed that part of chromosome 21q (green) moved onto chromosome 4p: der(4)t(4p;21q). **D**: The hybridization of a combination of DNA probes corresponding to WHS (red) on chromosome 4p and whole chromosome painting probe on chromosome 6p (green) revealed that WHS on chromosome 4p moved onto chromosome 6p: der(6)t(4p;6p). **E**: The hybridization of whole chromosome painting probe of chromosome 6 (red) and chromosome 21 (green) revealed that part of chromosome 6p moved onto chromosome 21q: der(21)t(6p;21q). [Color figure can be viewed in the online issue, which is available at www.interscience.wiley.com.]

Cytogenetic analysis and fluorescence in situ hybridization (FISH) with selected probes identified an abnormal male karyotype with an apparently balanced translocation 46,XY,t(4;6;21)(p16;p21.1;q21) (Fig. 3). Both parents had normal karyotypes. FISH analysis was performed on metaphase cells with a combination of whole chromosome painting probe 6 and a Wolf–Hirschhorn syndrome (WHS) locus-specific probe on chromosome 4p16.3. Locus-specific probes for chromosome 21 (LSI21, 21q22) were also used. A locus-specific probe, *AML1*, a subtelomeric probe of 21q, and whole chromosomal paint probes of chromosomes 4 and 21 were also used on the father's peripheral blood sample. All the probes were purchased from a commercial source (Vysis, Downers Grove, IL) and used according to the manufacturer's protocols with minor modifications. BAC, PAC, and fosmid clones spanning the 6p21 region were selected from the UCSC genome database (http://genome.ucsc.edu) and obtained from the BACPAC Resource Center (http://www.chori.org/bacpac).

**Fig. 4:**
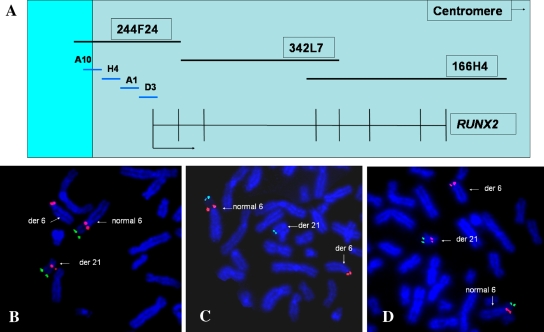
**A**: Schematic representation of the *RUNX2* genomic region, and relative location of the probes used for FISH mapping of the translocation breakpoint on 6p21. These include clones RP1-244F24 (AL096865), RP11-342L7 (AL358135), and RP1-166H4 (AL161907), and fosmids G248P80212A10, G248P86676H4, G248P8418A1, and G248P86565D3 located upstream of the *RUNX2* gene. **B**: FISH with probe RP1-244F24 (red) showed signals on the normal and the derivative chromosome 6 and the derivative chromosome 21. A telomeric probe for 6p (green) was used in B, C, and D. FISH was performed with fosmids located upstream of *RUNX2*: (**C**)Fosmid Probe G248P86676H4 (red) was only seen on the normal and derivative chromosome 6 (not translocated); (**D**) Fosmid G248P80212A10 (red) showed a split signal, being present on the normal chromosome 6 and both derivative chromosomes 6 and 21. [Color figure can be viewed in the online issue, which is available at www.interscience.wiley.com.]

Given the facts that the patient had a CCD phenotype and that the translocation involved the genomic location of *RUNX2*, we performed FISH analysis with three probes spanning the *RUNX2* locus. We used clones RP1-244F24 (AL096865), RP11-342L7 (AL358135), and RP1-166H4 (AL161907), which cover the promoter and entire coding region of the gene (Fig. 4a). Probes RP11-342L7 and RP1-166H4 showed signals only on the normal and derivative chromosome 6, and probe RP1-244F24 showed signals on the normal and derivative chromosome 6 as well as on chromosome 21. In order to refine the breakpoint on the derivative chromosome 6, we performed FISH using overlapping fosmids G248P80212A10, G248P86676H4, G248P8418A1, and G248P86565D3 located upstream of the *RUNX2* gene (Fig. 4a). Fosmid clones G248P86676H4, G248P8418A1, and G248P86565D3 located immediately upstream of the *RUNX2* gene produced hybridization signal on the normal and derivative chromosome 6 only, suggesting that the entire coding sequence of *RUNX2* including its promoter remained intact on the der(6) chromosome and was not translocated (Fig. 4b). Signals from fosmid G248P80212A10 were seen on the normal chromosome 6 and both derivative chromosomes 6 and 21 (breakpoint clone). This result maps the breakpoint on chromosome 6p21 to a region approximately 50 kb upstream of the *RUNX2* gene based on the genetic mapping position of G248P80212A10 and *RUNX2* on the UCSC genome website.

To exclude the possibility of a de novo mutation in the *RUNX2* gene, we sequenced DNA fragments amplified by PCR. The *RUNX2* exons and promoter regions were amplified using intron- and exon-specific primers as described previously [Lee et al. 1997, Napierala et al. 2005]. Results were normal.

To determine if small deletions or duplication around translocation breakpoints may have contributed to the atypical findings seen in this patient, we screened for cryptic copy number variants by oligonucleotide-based array comparative genome hybridization (aCGH). aCGH was performed using a Human Genome CGH 244A Oligo Microarray Kit (Agilent Technologies, Inc., Santa Clara, CA) according to the manufacturer's protocol version 4.0. Arrays were scanned using an Agilent DNA Microarray Scanner and data were extracted using Feature Extraction Software 9.1 and analyzed using CGHAnalytics 3.4.27 Software (Agilent Technologies, Inc., Santa Clara, CA). DNA from a healthy male with no personal or family history of CCD served as a control. A duplication of <77 kb was detected distal to the *BAK1* gene on chromosome 6p21.3. This region is devoid of any known genes. All other copy number changes identified in the regions around 4p16, 6p21, and 21q21 were within copy number variant regions previously described in the Database of Variants hosted by the Centre for Applied Genomics (http://projects.tcag.ca/variation/) or represented changes in the HLA gene clusters on chromosome 6. No obvious pathologic duplications or deletions were found within the remainder of the genome.

## DISCUSSION

CCD is a well-defined clinical phenotype arising from deregulation of intramembranous and endochondral ossification. The majority of cases are due to loss of function mutations in the *RUNX2* gene that encodes for a transcription factor essential for osteoblast differentiation and chondrocyte maturation. Microdeletions and translocations involving *RUNX2* explain an additional fraction of the patients. For proper skeletal development and homeostasis, the expression of *RUNX2* is tightly regulated during development in a strict temporal and spatial fashion. This regulation is achieved through regulatory regions including two promoters [Napierala et al., 2005]. The mouse *RUNX2* gene also contains two separate promoters [Xiao et al., 1998] and, in osteoblasts, *RUNX2* expression is driven by the distal bone-related promoter. Napierala et al. 2005 reported two patients with promoter sequence variants and hypothesized that promoter mutations affecting binding of transcription factors critical for *RUNX2* expression might change the transcriptional activity of the gene.

Although the main regulation of *RUNX2* expression is driven by two promoters, the promoter sequence alone does not seem to be sufficient for adequate control of the expression. Additional *cis*-acting regulatory sequences located 200 and 400 kb upstream of exon 1 have been identified that act as enhancers of the *RUNX2* gene [Stock and Otto, 2005]. The translocation of the *RUNX2* gene on 6p to chromosome 21 in our patient may have disrupted a 5′-control element, such as an enhancer, resulting in haploinsufficiency for *RUNX2* and CCD phenotype. Given that in our patient the breakpoint was 50 kb upstream of exon 1 of the *RUNX2* gene, this case suggests the location of a novel *RUNX2* regulatory element in this region.

A patient with a de novo reciprocal translocation t(6;7)(p21.1;q36) with CCD and holoprosencephaly has been reported [Fernandez et al., 2005]. The authors proposed that the phenotype was due to two position effect mutations, one at each breakpoint, altering the expression of the *SHH* (holoprosencephaly) and *RUNX2* (CCD) genes. CCD-like phenotypes have also been associated with de novo cytogenetically balanced translocations: t(2;6)(q36;q16) [Winer et al., 2003], and t(6;18)(p12;q24) [Narahara et al., 1995]. A male patient with a pericentric inversion of chromosome 6 and classic CCD with mild to moderate mental retardation, hearing deficits, and unusual facial appearance has also been reported [Nienhaus et al., 1993].

Atypical features seen in this patient cannot be explained by dysregulation of *RUNX2* expression. The patient's developmental delay, easy bruising, and features of connective tissue disorder are not characteristic of CCD. We hypothesize that disruption of additional genes on the other two chromosomes involved in the translocations (4 and 21) contribute to this patient's complex phenotype. The possibility of microdeletions detectable by array CGH has been excluded; however, the breakpoints could have disrupted the coding sequence of another gene, or resulted in positional effects affecting other genes. Chromosome region 4p16 has the critical genes that, when deleted, result in WHS (OMIM 194190), characterized by failure to thrive, mental retardation, distinct facial features, and seizures. Although our patient does not have characteristics suggestive of WHS, it is possible that a gene involved in cognitive development could have been affected. A gene involved in neural adhesion, *NCAM2* has been mapped to chromosome region 21q21.

Position effect mutations may result in disease by repositioning genetic material and thereby leading to altered gene expression in the absence of an intragenic change [Fernandez et al., 2005]. A chromosome rearrangement may separate a gene from distal regulatory elements or exert its effects on chromatin structure. We propose that our patient's CCD phenotype is caused by disruption of a regulatory element for the *RUNX2* gene at the 6p21 breakpoint causing altered expression of the gene resulting in CCD. Further studies might unravel a new regulatory element for *RUNX2*.
